# Smart Home Automation-Based Hand Gesture Recognition Using Feature Fusion and Recurrent Neural Network

**DOI:** 10.3390/s23177523

**Published:** 2023-08-30

**Authors:** Bayan Ibrahimm Alabdullah, Hira Ansar, Naif Al Mudawi, Abdulwahab Alazeb, Abdullah Alshahrani, Saud S. Alotaibi, Ahmad Jalal

**Affiliations:** 1 Department of Information Systems, College of Computer and Information Sciences, Princess Nourah bint Abdulrahman University, P.O. Box 84428, Riyadh 11671, Saudi Arabia; bialabdullah@pnu.edu.sa; 2 Department of Computer Science, Air University, E-9, Islamabad 44000, Pakistan; hiraansar53@gmail.com; 3 Department of Computer Science, College of Computer Science and Information System, Najran University, Najran 55461, Saudi Arabia; afalazeb@nu.edu.sa; 4 Department of Computer Science and Artificial Intelligence, College of Computer Science and Engineering, University of Jeddah, Jeddah 21589, Saudi Arabia; asalshahrani2@uj.edu.sa; 5 Information Systems Department, Umm Al-Qura University, Makkah 24382, Saudi Arabia; ssotaibi@uqu.edu.sa

**Keywords:** feature fusion, filter, home automation, adaptive median filter, hand detection, deep learning, noise reduction, gesture recognition

## Abstract

Gestures have been used for nonverbal communication for a long time, but human–computer interaction (HCI) via gestures is becoming more common in the modern era. To obtain a greater recognition rate, the traditional interface comprises various devices, such as gloves, physical controllers, and markers. This study provides a new markerless technique for obtaining gestures without the need for any barriers or pricey hardware. In this paper, dynamic gestures are first converted into frames. The noise is removed, and intensity is adjusted for feature extraction. The hand gesture is first detected through the images, and the skeleton is computed through mathematical computations. From the skeleton, the features are extracted; these features include joint color cloud, neural gas, and directional active model. After that, the features are optimized, and a selective feature set is passed through the classifier recurrent neural network (RNN) to obtain the classification results with higher accuracy. The proposed model is experimentally assessed and trained over three datasets: HaGRI, Egogesture, and Jester. The experimental results for the three datasets provided improved results based on classification, and the proposed system achieved an accuracy of 92.57% over HaGRI, 91.86% over Egogesture, and 91.57% over the Jester dataset, respectively. Also, to check the model liability, the proposed method was tested on the WLASL dataset, attaining 90.43% accuracy. This paper also includes a comparison with other-state-of-the art methods to compare our model with the standard methods of recognition. Our model presented a higher accuracy rate with a markerless approach to save money and time for classifying the gestures for better interaction.

## 1. Introduction

In recent years, home automation has emerged as a research topic. Many researchers have started investigating the demand criteria for home automation in different environments. Human–computer interaction (HCI) [1] is considered a more interactive and resourceful method of engaging with different appliances to make the system work. In the conventional approach, different devices like a mouse, keyboard, touch screen, and remote devices are used to fulfill requirements so that users can interact by only using their hands with different home appliances, home healthcare, and home monitoring systems. Usually, changing channels and controlling light on/off switches are more demanding research areas for HCI [2]. Earlier systems were divided into two approaches for interacting with computers. The first approach is inertial sensor-based and the second approach is vision-based. In the first approach, sensors are built with one or more arrays. They track the position of the hand, the velocity, and acceleration. Then, these motion features are trained and tested for hand gesture recognition. They are used to control home appliances like TV, radio, and lights [3,4,5,6,7]. Despite its high sensitivity, this approach makes it difficult to obtain higher accuracy. This approach demands a proper setup with high-quality sensors. The use of high-quality sensors can attain better results, but they make the system more expensive, and durability issues arise. With the advancement of technology, new sensors are continually being launched in the market [8], the purpose of which is to minimize sensitivity, making them more expensive.

The second approach is vision-based, which reduces the limitations arising from the sensor-based approach [9]. With the help of this sensor, hand gestures are recognized using images. The images consist of RGB and depth. The RGB images are collected using cameras. The cameras are less expensive and easy to set up properly. The RGB image color, shape, orientation, contours, and positions are calculated for hand gesture recognition. The vision-based sensors with depth images gain more dimensions than RGB [10]. For depth, thresholding techniques are either empirical or automated. Empirical techniques include the trial-and-error method, in which the search space is excluded, and the computation cost is a priority for hand localization. In automated solutions, the hand is considered the main focus area for data acquisition [11]. The hand is localized as the closest object in front of the camera’s in-depth image.

Vision-based sensors also pose some challenges for researchers, such as light intensity, clutter sensitivity, and skin tone color [12]. Hand localization is a crucial step. For this, the conventional systems are divided into different steps to obtain better accuracy while keeping the challenges in view. First, data acquisition is performed, followed by hand detection. For hand detection, multiple methods are used, including segmentation, tracking, and color-based extractions. The features are extracted using different algorithms. After that, the gesture is recognized. For the given approach, both images and videos are collected [13]. The still images provide static gestures, whereas videos provide dynamic hand gestures, as changes in hand gestures from one frame to another are noticed. Static gestures are still images and require less computation cost [14,15,16,17], whereas dynamic gestures contain three-dimensional motion. The movement in dynamic hand gestures becomes a challenging task as the speed varies, and gesture acquisition is difficult due to speed issues. In the literature, static and dynamic gesture recognition has been performed using two different methods: supervised and unsupervised learning. Supervised learning methods include decision trees, random forests, and SVM, whereas unsupervised learning methods include k-means, hidden Markov model, and PCA [18].

In our proposed model, we have used dynamic gestures to challenge our limitations. Our system proved its compatibility. In this paper, the videos are first converted into frames. An adaptive median filter and gamma correction are applied to the images to reduce noise and adjust the light intensity, respectively. Then, the hand is detected using saliency maps. The extracted hand is then available for feature extraction. We have extracted different features while keeping the issues hindered in classification. For this feature, we have chosen three different state-of-the-art algorithms. These features are named the joint color cloud, neural gas, and directional active model. The features are then optimized using an active bee colony algorithm. The optimized features are passed through the RNN. Our accuracies are shown to be better for model designs. The main contributions of our system are as follows:

- The system approach is different from previous systems; it recognizes dynamic gestures with complex backgrounds.
- Hands are detected from both images using two-way detection: first, the skin tone pixels are extracted, and then the saliency map is applied for greater precision.
- Features are collected using different algorithms, like fast marching, neural gas, and the 8-freeman chain model. All the features are extracted with modifications to the algorithms listed. The features are collected and fused to make a feature fusion for recognition.
- The proposed system uses a deep learning algorithm such as RNN to achieve higher accuracy.

The rest of the sections presented in this article are as follows: Section 2 includes a related study of the existing methods. Section 3 presents the architecture of the proposed system. Section 4 shows the experimental section with system performance evaluations. Section 5 describes the strengths and weaknesses of our proposed system. Section 6 presents the conclusion of the system and future work directions.

## 2. Literature Review

Multiple methods have been introduced to acquire hand gestures. This section presents the most useful and popular methods. A literature review was conducted to study the research work carried out in particular areas.

### 2.1. Hand Gesture Recognition via RGB Sensors

In hand gesture recognition systems, many researchers use sensors and cameras to recognize gestures. The RGB videos can be collected using different cameras. Table 1 presents the methods used by researchers for hand gesture recognition using RGB videos.

### 2.2. Hand Gesture Recognition via Marker Sensors

Many researchers worked on marker sensors with proper equipment setup. Gloves were attached to the hands to note down the locations and movements. Table 2 presents the researchers’ methods for hand gesture recognition using marker videos.

## 3. Materials and Methods

### 3.1. System Methodology

**Figure 1:**
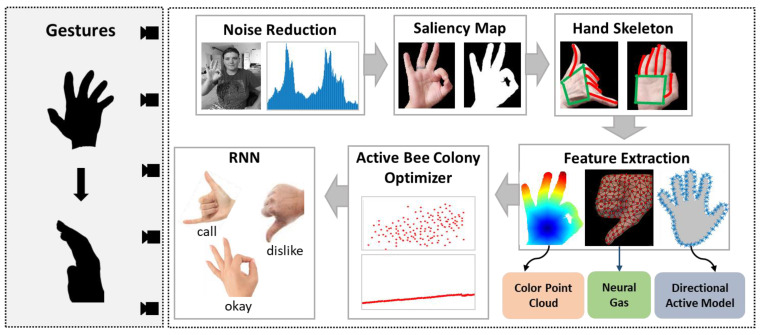
Architecture of the proposed system for hand gesture recognition.

The proposed architecture detects hand gestures in a dynamic environment. Primarily, for a dynamic image, the images are first converted into frames. The acquired images are passed through an adaptive mean filter for noise reduction, and then gamma correction is applied to the images to adjust the image intensity for better detection. On the filtered images, skin color is detected, and a saliency map is applied over it for hand extraction. The extracted hand is trained over a pre-defined model for the hand skeleton. After that, the detected hand and skeleton are used for feature extraction. The features include a joint color cloud, neural gas, and a directionally active model. The features are optimized to reduce complexity via graph mining. Finally, for the gestures, an RNN is implemented for classification. The architecture of the proposed system is presented in Figure 1.

### 3.2. Images Pre-Processing

In the acquired image, noise reduction is necessary to remove extra pixel information, as extra pixels hinder detection [38,39,40,41]. An adaptive median filter is used to detect the pixels affected by noise. This filter maintains the image quality, and the image blurring effect is negated. The pixels in the noised image are compared with the values of their neighboring pixels. A pixel showing a dissimilar value is labelled as a noisy pixel and a filter is applied over it. The pixel value is adjusted and replaced with the value of its neighboring pixels. For every pixel, the local region statistical estimate is calculated, resulting in $\hat{a}$; a is the uncorrupted image, and $\hat{a}$ is obtained from this image. The mean square error (MSE) is minimized between these two images, $\hat{a}$ and a. The MSE is presented as follows:

(1) $$m^{2}=Ea-{\hat{a}}^{2}$$

Conventional filters change all pixel values to denoise the image, but adaptive median filters work in two ways to change only the dissimilar pixels. Between level A and level B, level A is presented as follows:

(2) $$\begin{array}{c} A1=Q_{med}-Q_{min}\\ A2=Q_{med}-Q_{max} \end{array}$$

where $Q_{med}$ represents the median of the gray level in the original image $I_{xy}$; $Q_{min}$ is the minimum gray level in $I_{xy}$; $Q_{max}$ is the maximum gray level in $I_{xy}$. If A1>0 and A2<0, there is a shift to level B. Otherwise, the window size is increased if the window size is less than or equal to $I_{max}$ repeat level A, whereas $I_{max}$ represents the maximum size of $I_{xy}$. Otherwise, the gray level coordinates $Q_{xy}$ are shown. Level B is presented as follows:

(3) $$\begin{array}{c} B1=Q_{xy}-Q_{min}\\ B2=Q_{xy}-Q_{max} \end{array}$$

**Figure 2:**
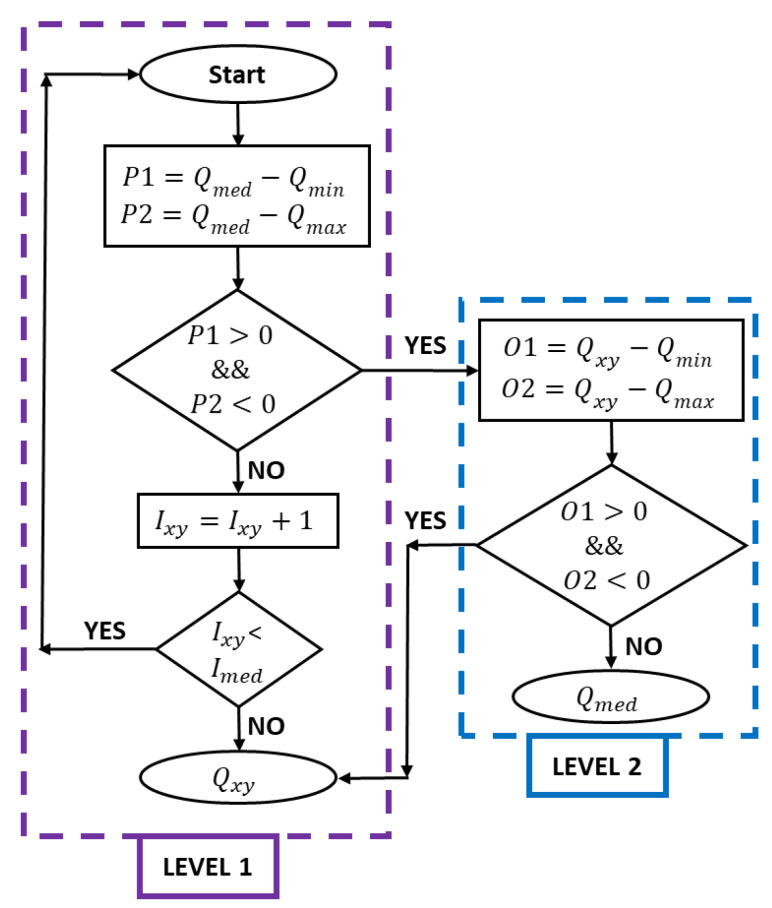
Sequential model representation for adaptive median filter algorithm.

If B1>0 and B2<0 then $Q_{xy}$ is shown, otherwise $Q_{med}$ is shown. Figure 2 shows a flowchart of the algorithm implemented for the filter.

**Figure 3:**
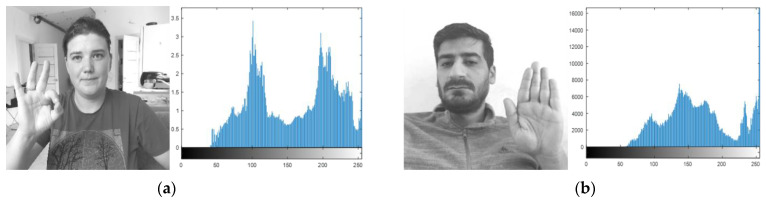
Pre-processed images with histograms of HaGRI dataset gestures: (**a**) ok; (**b**) stop.

The denoised image intensity is adjusted via gamma correction, as brightness plays a key role in the detection of a region of interest [42]. The power law for gamma correction is defined as follows:

(4) $$W_{o}=GW_{I}^{\gamma }$$

where $W_{I}$ is the input non-negative value with power γ and G is the constant usually equal to 1, and the range can lie between 0 and 1. $W_{o}$ is the output value [43,44,45]. The denoised intensity-adjusted image, including the plot, is shown in Figure 3.

### 3.3. Hand Detection

**Figure 4:**
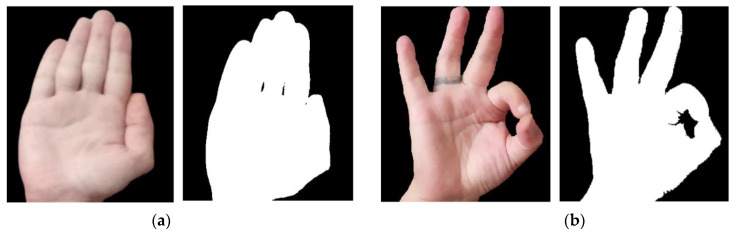
Hand detection using saliency map on the gestures (**a**) stop and (**b**) ok.

In this section, the hand is detected from the images using a two-way model. First, the skin tone from pixels is detected using hand gestures to localize the region of interest [46,47,48,49,50]. Then, a saliency map is applied over the image to obtain a better view of the desired gesture. The saliency map goal is to find the appropriate localization map, which is computed as follows:

(5) $$\begin{array}{c} M_{s\;}^{h}\in \;{\mathbb{R}}^{u*v}=ReLU\;\left(\sum_{i}\alpha_{i}^{h}H^{i}\right)\\ \alpha_{i}^{h}=\frac{1}{R}\left(\underset{i}{\sum }\underset{j}{\sum }\right) \end{array}$$

where $M_{s\;}^{h}$ is the localization map for the region of interest; u∗v represents the width and height of the image; i is the region of interest; $\alpha_{i}^{h}$ represents the global average pooling; R is the gradient via backpropagation. The average of the feature map is calculated using the weights assigned to the pixel gradient. Then, the ReLU is applied over the feature map. The image view range is set between 0 and 1, and the image is upscaled and overlay on the original image, resulting in a saliency map [51,52,53]. Figure 4 presents the saliency map for the HaGRI dataset “stop” and “ok” gestures.

### 3.4. Hand Skeleton

**Figure 5:**
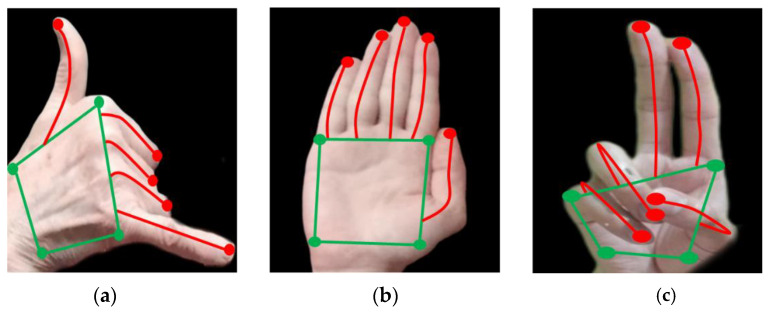
Hand skeleton mapping presenting palm and finger extreme points over gestures: (**a**) call; (**b**) stop; (**c**) two up.

For hand skeleton mapping, hand localization is the foremost step [54]. In our research, we first separated the palm and fingers for an accurate classification of the skeleton points. For palm extraction, a single-shot multibox detector (SSMD) is used; it excludes the fingers, and only the palm is bound by the blob. Then, the palm is first converted into binary, and a four-phase sliding window is moved across the whole area for the detection of the four extreme left, right, top, and bottom points. The second phase of the system includes finger identification; again, SSMD is used to detect the fingers. The palm is excluded, and the four-phase sliding window is moved to the extracted fingers again. It identified the extreme top, bottom, left, and right points [55]. From the extreme tops, the curves of the pixels are noted and marked. As a result, five points on the fingers and four points on the palm are obtained. Figure 5 shows the hand skeleton results for the HaGRI dataset.

### 3.5. Fusion Features Extraction

In this section, we illustrate how to extract various features from the acquired hand gestures. In hand gesture recognition systems, feature extraction contains two types of features: full-hand and point-based [56]. The full-hand feature set is made up of two techniques: a joint colour cloud and neural gas. A directionally active model is included in the point-based feature. Both the extracted features are fused together to generate a feature set for recognition.

#### 3.5.1. Joint Color Cloud

For this feature, the algorithm used to generate the cloud with different colors, which helps to obtain the skeleton point accuracy, and the geodesic distance for all fingers, including the palm, is extracted for the feature set. The color cloud is generated using a fast-marching algorithm [56,57,58]. This algorithm is defined as follows:

(1) Suppose we are interested in the region of interest function value f(i,j). This leads to two types of spatial derivative operators.
  (6) $$\begin{array}{c} S^{+i}f=\frac{f\left(i+t,\;j\right)-f\left(i,j\right)}{t}\\ S^{-i}f=\frac{f\left(i,j\right)-f\left(i+t,\;j\right)s}{t} \end{array}$$
  where $S^{+i}f$ is the forward operator, as it uses the f(i+t, j) to propagate from right to left by finding the value of f(i,j). On the other hand, $S^{-i}f$ represents the backward operator, propagating from left to right.
(2) For the difference operator, a discrete function is used to calculate $f_{i,j}$. For this purpose, at a specific point, the speed function $P_{i,j}$ is defined as follows:
  (7) $$\;{\left[\begin{array}{c} \max{\left(S_{i,j}^{-i}f,-S_{i,j}^{+i}f,\;0\right)}^{2}\\ +\max{\left(S_{i,j}^{-j}f,-S_{i,j}^{+j}f,\;0\right)}^{2} \end{array}\right]}^{\frac{1}{2}}=\frac{1}{P_{i,j}}$$
  The above equation is interpreted as follows, where (i,j) is the arrival time of $f_{i,j}$.
  (8) $${\left[\begin{array}{c} \mathrm{Max}{\left(f_{i,j}-f_{i-1,j},\;f_{i,j}-f_{i+1,j},\;0\right)}^{2}\\ +\max{\left(f_{i,j}-f_{i,j-1},\;f_{i,j}-f_{i,j+1},\;0\right)}^{2} \end{array}\right]}^{\frac{1}{2}}=\frac{1}{P_{i,j}}$$
(3) For the neighbor pixel value calculation, only $f_{i,j}$ point included in the set point (i,j) can be used. The $f_{i,j}$ value computation is defined as follows:
  (9) $$\begin{array}{c} p=\min\left(f_{i-1,j},\;f_{i+1,j}\right)\\ q=\min\left(f_{i,j-1},\;f_{i,j+1}\right) \end{array}$$
(4) The quadratic equation is formulated for $f_{i,j}$: $\mathrm{if}\;\frac{1}{f_{i,j}}>\left|p-q\right|,$ which leads to the following:
  (10) $$\;f_{i,j}=\frac{p+q+\sqrt{2{\left(\frac{1}{f_{i,j}}\right)}^{2}-{\left(a-b\right)}^{2}}}{2}$$
  $$otherwise,\;f_{i,j}={\left(\frac{1}{f_{i,j}}\right)}^{2}+\min\left(a,b\right)\;$$

**Figure 6:**
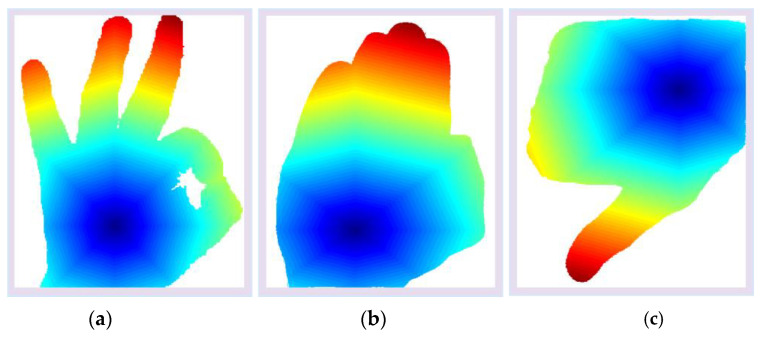
Wave propagation of point-colored cloud over HaGRI datasets gesture. (**a**) ok; (**b**) stop; (**c**) dislike.

These computations have only been performed on the neighbors of the new points added. If the neighboring value and calculated point (i,j) are equal then the values are compared, and the smaller value calculated before is added. In every iteration, a smaller value is found and stored. To save time, the min heap is used in the fast-marching algorithm to store the minimum values quickly with less time consumption. These iterations continue until the endpoint is achieved [59,60]. Figure 6 shows the results for the point-colored cloud.

#### 3.5.2. Neural Gas

**Figure 7:**
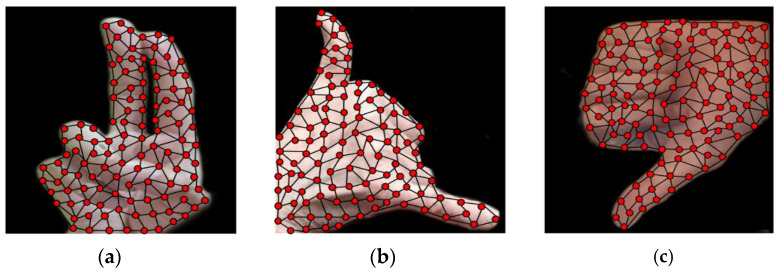
Neural gas formation with self-organized shape for gestures:(**a**) two up; (**b**) call; (**c**) dislike.

Neural maps organize themselves and form neural gas; it shows the ability to rank neighborhood vectors, which determine the neighborhood data space [61,62]. The neural gas is composed of multiple neurons, n, comprising weight vectors  W(r) that result in forming clusters. During training, every single neuron presents a change in position with an abrupt movement. Randomly, a feature vector is assigned to every single neuron. From the formed neural gas network, random data r is selected from the feature vector. With the help of this data vector r, the Euclidean distance is calculated from all the weight vectors. The distance values computed determine the center adjustment with the selected data vector [63,64,65]. The feature vector itself is defined as follows:

(11) $$\begin{array}{c} W_{f_{n}}^{m+1}=W_{f_{n}}^{m}+\varepsilon .\;e^{-n/⋋}.\;\left(r\;–\;W_{f_{n}}^{m}\;\right),\\ n=0,\;\ldots .\;,\;N-1 \end{array}$$

where the probability distribution W(r)  of the data vector n with a finite number of sets $s_{f},\;f=1,\;\ldots ..,\;N.\;$ A data vector n for probability distribution W(r) is presented at each time step m. The distance order is determined from the feature vector of the given data  r. If $n_{o}$ is the index of the closed feature vector, $n_{1}\;$ is the second, and $n_{N-1}$ is distant to the data vector n, then ε represents adaptation step size and ⋋ represents neighborhood range. After most of the adaptation steps, the data space is covered with a feature vector with minimum errors. Algorithm 1 defines the pseudocode for neural gas formation, and Figure 7 presents the structure of the neural gas over the HaGRI dataset gesture.
**Algorithm 1: Pseudocode for Neural Gas Formation****Input**: I: Input space;**Output**: $G=\left(n_{0},n_{1},\;\ldots ..,n_{N}\right):$ the map;I← []**Method**:I← $N\left(n_{0},n_{1}\right)$, where $n_{0}$ represents the first node and $n_{1}$ represents the second node$n_{0}$← 0;$n_{N}$← 100;**Whereas**, the input signal Φ is as follows:     I← [Φ]     Calculate winning nodes nearest Φ             
$p_{1\;}\leftarrow argmin_{o\;\in O}\;∥\;\Phi -w_{n}∥$
             
$p_{2}\leftarrow argmin_{o\;\in \left\{p_{1}\right\}}\;∥\Phi -w_{n}∥$
      Adjust $p_{1\;}\mathrm{and}\;p_{2}\;$
       
$edge\leftarrow \;edge\;\cup^{\;}\left(p_{1\;},\;p_{2}\right)\;$
       
edge←0
$\;\;\;\;\;\;\;\;\;\;\;\;{\;\mathrm{error}\;}_{p_{1}}\leftarrow \;{\;\mathrm{error}\;}_{p_{1}}+∥\;\Phi -w_{p_{1}}∥$         Adjust edge
           I← [$n_{i+1}$]    **repeat****until** $n_{N}$← 100**end while****return** $G=\left(n_{0},n_{1},\;\ldots ..,n_{N}\right)$

#### 3.5.3. Directional Active Model

**Figure 8:**
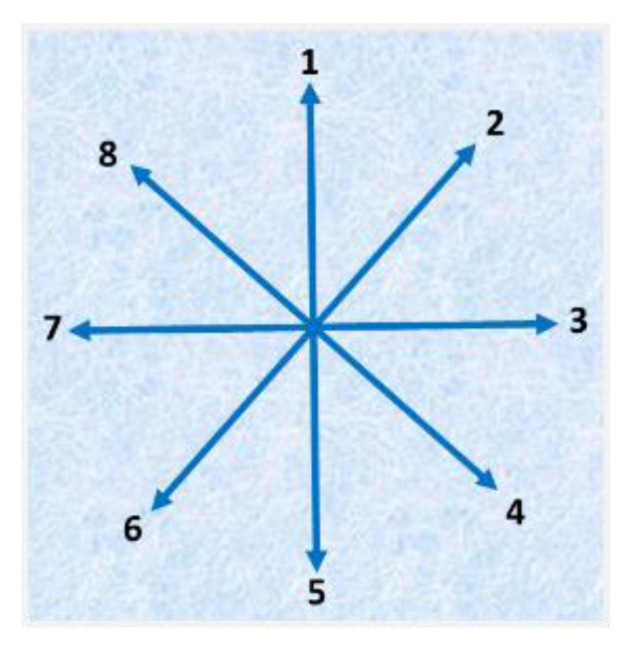
Direction representation of eight Freeman chain codes.

The next feature is extracted using an 8-Freeman chain code algorithm, which measures the change in the directions of the curves at the boundary of the hand gesture [66]. Eight Freeman chain codes are shape descriptors, and they change structural schemes with a contour-dependent scheme. A shape description possesses a set of lines oriented in a particular manner. The oriented vectors are in eight and four directions, and the chain code vectors have integer numbers represented in a possible direction, as shown in Figure 8.

**Figure 9:**
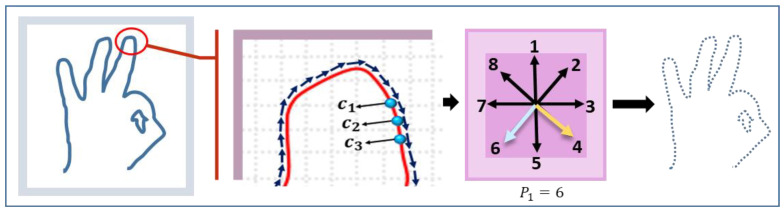
Flow sheet of point extraction in a directionally active model.

First, the boundary of the hand is identified to obtain the curves. Suppose the points on the curve are denoted by c on the boundary d. The starting point t on the top-right side of the thumb orientation is checked for its vector position. The curve points on the boundary $P_{b}$ are calculated for all points, so it becomes $P_{b}=\left\{t_{0},t_{1},\;\ldots \ldots ,\;t_{n-1}\right\}.$ After attaining the vector position of $t_{0}\;\mathrm{and}\;t_{1}$, both of the curve point directions are compared; if they both have the same values, the value of $t_{1}$ is not considered and the next point $t_{2}$ vector position is checked; otherwise, both of the curve point values are added to the list. Hence, this whole procedure continues until $t_{n-1}$ is reached. Figure 9 depicts the flow of the point extraction in a directionally active model.

**Figure 10:**
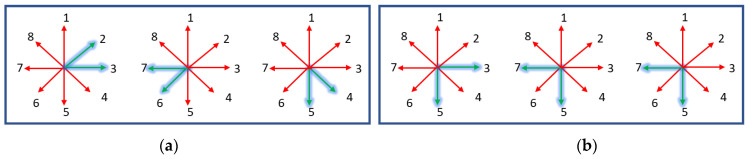
Angle demonstration for active directional model feature sets: (**a**) 45°; (**b**) 90°.

For our proposed system feature vector, we considered only 12 positions: 8 with an angle of 45° and 5 with an angle of 90°. The angle description is shown in Figure 10, which illustrates a better demonstration of the feature vector [67].

### 3.6. Feature Analysis and Optimization

**Figure 11:**
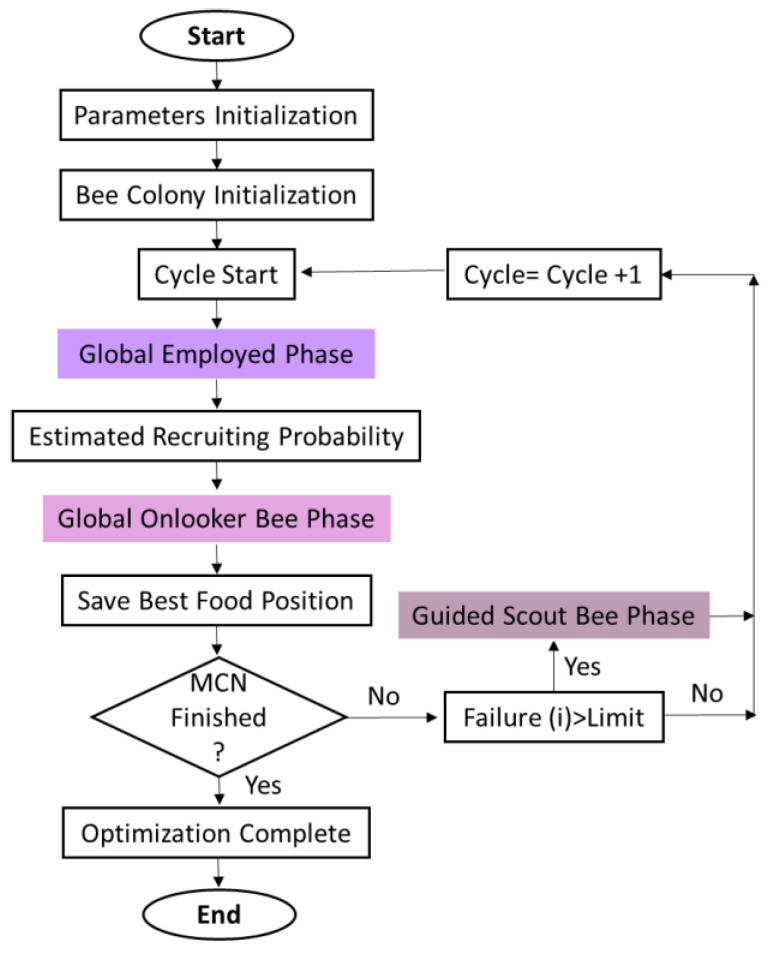
Flowchart of a working model for ABCA.

**Figure 12:**
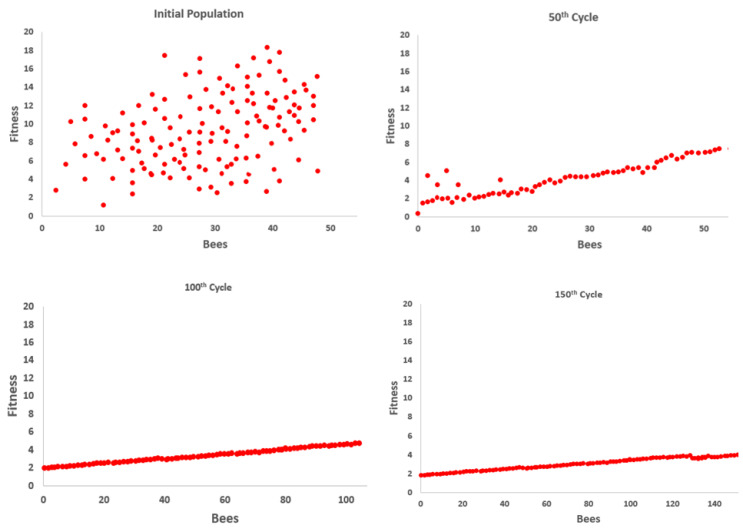
Angle fitness along the number of iterations over the “call” gesture.

After feature extraction from all datasets, the extracted features are passed through an artificial bee colony algorithm (ABCA) for optimization [68]. This helps reduce the computation time and also the complexity of the data. ABCA consists of two groups: one is known as the employer bee and the other is the onlooker bee. Both groups of bees have the same number, which is similar to the solutions in the group of honey bees, known as a swarm. The swarm size generates a randomly distributed initial population. Suppose the number of j-th solutions in the swarm is denoted as $X_{j}=\left(x_{j,1},x_{j,2},\;\ldots ..,x_{j,n}\right)$. Employed bees find their food sources as follows:

(12) $$a_{j,i}=x_{j,i}+\emptyset_{j,i}\;.\;(x_{j,i},\;x_{j,l})$$

where $X_{l}$ represents the candidate solution and is randomly selected when j≠l. $\emptyset_{j,i}$ represents a random number from the range [–1, 1]. l is the dimension index from {1,2,3,…N}. When the food search by employee bees is completed, they share all the information between the onlookers and nectar. Then, they choose the food amount equal to the nectar amount. The fitness function of the new candidate solution is defined as follows:

(13) $$Pn_{j}=\frac{fit\left(j\right)}{\sum_{j=1}^{N}fit\left(j\right)}$$

where $Pn_{j}$ is the probability of the food source, which is higher if the solution better than j  is achieved. fit represents the fitness value in the j-th swarm size. With predefined function iterations, if the position is not changed, then the value of the food source $X_{j}$ is replaced with $X_{j,i}$ found by scout bees:

(14) $$X_{j,i}=kb_{i}+rand\left(0,1\right).\;(ob_{i}-kb_{i})$$

where $ob_{i}\;\mathrm{and}\;kb_{i}$ are the lower and upper boundaries of the i-th dimension; rand(0,1) represents the random values between 0 and 1, respectively. Figure 11 presents the overall flowchart of the artificial bee colony to determine the decision steps, while Figure 12 presents the best fitness result over the “call” gesture in the HaGRI dataset.

### 3.7. Gesture Classification Using RNN

**Figure 13:**
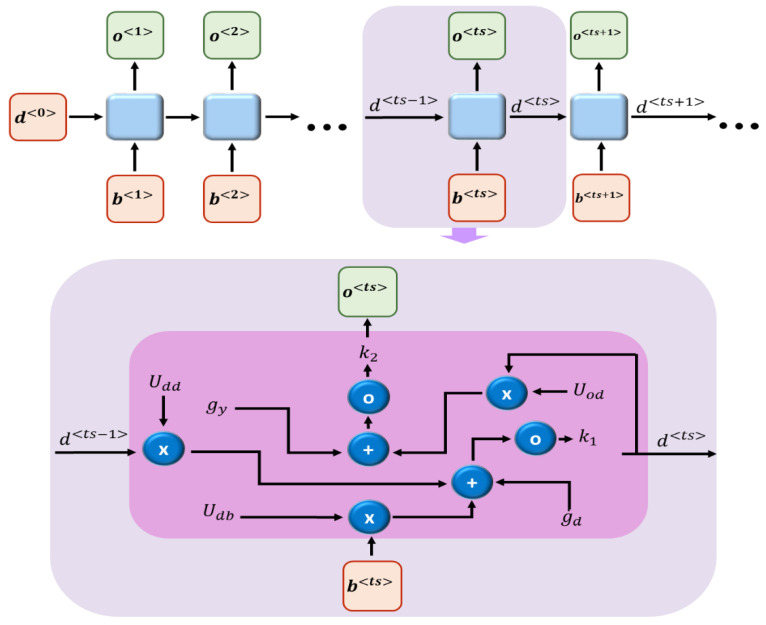
Overall flow of RNN architecture.

We used a recursive neural network (RNN) on our optimized feature vectors to classify gestures [69]. An RNN is a deep neural network that has the ability to learn distributive and structured data. Therefore, it is ideal for our proposed system of classification. In an RNN, the last output is typically used as the input for the next layer with hidden states. For each timestamp ts, the activation function $d^{\langle ts\rangle }$ and the output $o^{\langle ts\rangle }$ defined are as follows:

(15) $$d^{\langle ts\rangle }=k_{1}\left(U_{dd}d^{\langle ts-1\rangle }+U_{db}b^{\langle ts\rangle }+g_{d}\right)$$

(16) $$o^{\langle ts\rangle }=k_{2}\left(U_{od}d^{\langle ts\rangle }+g_{y}\right)$$

where $U_{dd},\;U_{db},\;U_{od,\;},\;g_{d},\;g_{y}$ are the coefficients shared temporarily. $k_{1},\;\;k_{2}$ are activation functions. Figure 13 presents the overall flow of the RNN architecture.

## 4. Experimental Setup and Evaluation

Experiments were performed on a system with the specifications of an Intel Core i7-9750H with 2.60GHz processing power, and 16GB RAM with ×64 based Windows 10. The MATLAB tool and Google Colab were used for attaining the results. The system accessed the performance of the proposed architecture on four benchmark datasets: HaGRI, Geogesture, Jester, and WLASL. The k-fold cross-validation technique was applied to all three datasets to verify the reliability of our proposed system. This section includes a dataset description, the experiments performed, and a system comparison with other state-of-the-art systems.

### 4.1. Dataset Descriptions

#### 4.1.1. HaGRI Dataset

**Figure 14:**
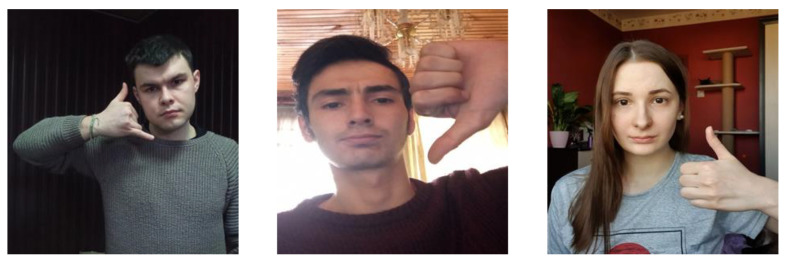
Example gesture frames from the HaGRI dataset.

The HaGRI Dataset [70] is specially designed for home automatic, automatic sector, and video conferencing. It consists of 552,992 RGB frames with 18 different gestures. The dataset includes 34,730 subjects who performed gestures with different backgrounds. The subjects were aged between 18 and 65 years old. The gestures were performed indoors with different light intensities. The gestures used in our experiments were call, dislike, like, mute, ok, stop, and two up. Figure 14 presents the gestures from the HaGRI dataset.

#### 4.1.2. Egogesture Dataset

**Figure 15:**
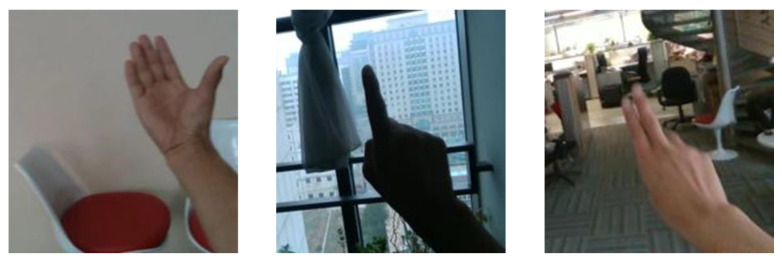
Example gesture frames from the Egogesture dataset.

The Egogesture [71] contains 2081 RGB videos and 2,953,224 frames with 83 different static and dynamic gestures. The gestures contain indoor and outdoor scenes. For our system training and testing, we selected seven dynamic gesture classes: scroll hand towards the right, scroll hand downward, scroll hand backward, zoom in with fists, zoom out with fists, rotate finger clockwise, and zoom in with fingers. The dataset samples with different gestures and different backgrounds are presented in Figure 15.

#### 4.1.3. Jester Dataset

**Figure 16:**
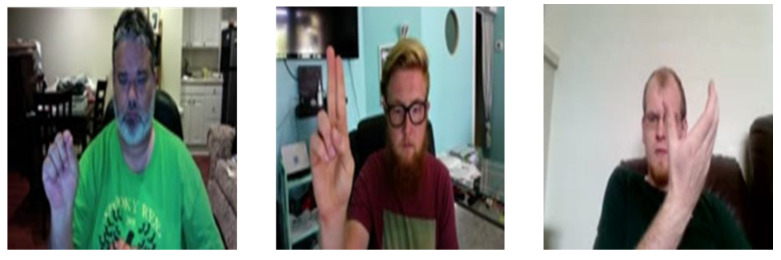
Example gesture frames from the Jester dataset.

The Jester dataset [72] contains 148,092 video clips of pre-defined human hand gestures collected in front of cameras; it comprises 27 gestures. The video quality of the gestures is set to 100 pixels at 12 fps. Seven hand gestures are selected for system training and testing for the following: sliding two fingers down, stop sign, swiping left, swiping right, turning the hand clockwise, turning the hand counterclockwise, and zooming in with two fingers. The example gestures of the Jester dataset are shown in Figure 16.

#### 4.1.4. WLASL Dataset

**Figure 17:**
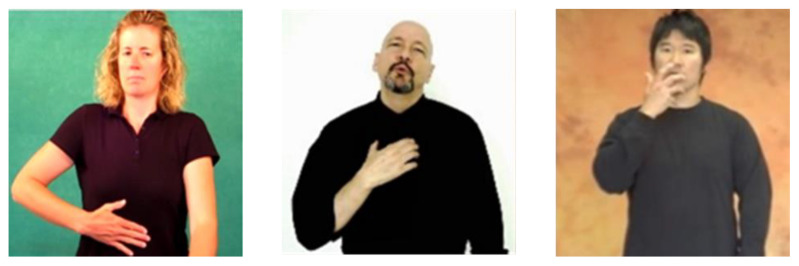
Example gesture frames from the WLASL dataset.

The WLASL dataset has the largest number of videos of American Sign Language hand gestures [73]. It has a total of 2000 hand gesture classes. The dataset was created specifically for communication between the deaf and hearing communities. We used seven classes to test the validity of our proposed model on hand gesture recognition datasets: hungry, wish, scream, forgive, attention, appreciate, and abuse. The WLASL dataset sample images are shown in Figure 17.

### 4.2. Evaluation via Experimental Results

**Figure 18:**
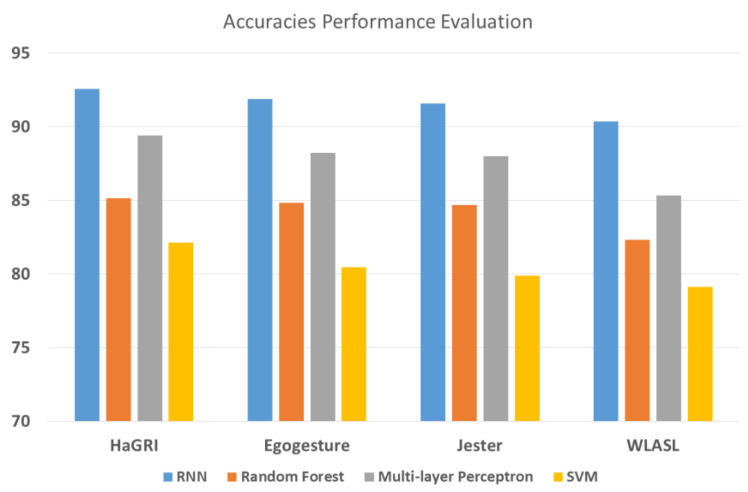
Accuracy comparison of RNN with other state-of-the-art algorithms.

We evaluated the performance of our proposed system on all three datasets, and the experiments proved the system’s efficiency. Table 3, Table 4, Table 5 and Table 6 illustrate the confusion matrices for the HaGRI, Egogesture, Jester, and WLASL datasets, achieving accuracy of 92.57%, 91.86%, 91.57%, and 90.43%, respectively. The experiments were repeated many times to evaluate the efficiency of the results. The HaGRI dataset presented the highest accuracy over the other datasets because of the higher resolution, and the hand extraction showed better results than the other datasets. Table 7, Table 8, Table 9 and Table 10 depict the gesture evaluation matrices for the HaGRI, Egogesture, Jester, and WLASL datasets. This presents the gesture class accuracy, precision, recall, and f1 score for all the benchmark datasets used. This section also compares the selected classifier’s accuracies to those of other conventional methods to demonstrate why they are preferred over other algorithms. Figure 18 demonstrates the comparison of the accuracy of RNN with other-state-of the art algorithms. Table 11 presents a comparison of our system with other conventional systems in the literature.

## 5. Discussion

The proposed hand gesture recognition system model is designed to achieve state-of-the-art performance over RGB images. Initially, images with a variety of gestures and complex backgrounds are used as inputs from benchmark datasets, such as HaGRI, Egogesture, and Jester. Our suggested two-way method is used to process the images provided for hand extraction. There were also some shortcomings in the proposed approach that prevented concealed information from being accurately obtained from the hand skeletons. Frames with no suitable camera angle made it difficult to acquire the exact key points at hand. As presented in Figure 5a, the extreme key points are localized on the knuckles of the fingers due to the absence of the fingertips in the frame. The suggested system performed well on frames that initially presented the entire hand, followed by the movement of the hand. After the hand and skeleton extractions, the region of interest was passed through the fusion of features. The full-hand and one-point-based features were optimized and passed through RNN for recognition. The accuracy attained over the four datasets via RNN produced better results, with an accuracy of 92.57% using the HaGRI dataset; for Egogesture, it was 91.86%; for Jester, it was 91.57%; and for WLASAL, it was 90.43%.

## 6. Conclusions

This paper provides a novel way of recognizing gestures in a home automation system. Home appliances like TVs, washing machines, lights, cleaning robots, printers, stoves, etc. can be controlled using hand gestures. Our system proposed a way to fulfill the requirement of detecting hands from a complex background via six steps, namely noise removal, hand detection, hand skeleton, feature extraction, optimization, and classification. The hand gestures were trained by preprocessing them first using the adaptive median algorithm. Then, the hand detection was performed using the two-way method, and after that, the hand skeleton was extracted using SSMD. From the extracted hand and skeleton points, fusion features were extracted, namely joint colour cloud, neural gas, and directional active model. The features were optimized using the active bee colony algorithm, which provided promising results for all four datasets. The accuracies attained using the HaGRI dataset was 92.57%; for Egogesture, it was 91.86%; Jester provided 91.57%; and WLASL showed 90.43%. The proposed system is for smart home automation, which was designed using different techniques. It provides a set of features for recognition, rather than conventional features, using only deep learning methods.

The proposed system needs to be trained with more gestures, and various experiments can be performed in different environments like healthcare, robotics, sports, and industries. The computation time needs to be considered to remove the complexity of the system. The computational cost of the system can be managed by considering the architecture. In the future, we plan to work under different circumstances using computational cost management. Also, we will add more features and robust algorithms to make our system more efficient and standard for all environments.

## Figures and Tables

**Table 1:** Related studies on hand gesture recognition using RGB sensors.

Authors	Methodology
S. Nagarajan et al. [19]	The proposed system captures the American sign language and filters the images using Canny edge detection. An Edge Orientation Histogram (EOH) for feature extraction was used, and these feature sets were classified by a multiclass SVM classifier; however, some signs were not detected due to hand orientation and gesture similarity.
Mandeep et al. [20]	The hand gesture system used the skin color model and thresholding; the YCbCr segmented the hand region, skin color segmentation was used to extract the skin pixels, and Otsu thresholding removed the image’s background. In the last PCA, the template-matching method was used to recognize a total of twenty images per gesture from five different poses from four gesture captures. On the other hand, this system has some limitations in that skin color varies due to light colors, and the background contains skin color pixels.
Thanh et al. [21]	Multimodal streams are used to increase the performance of hand recognition by combining depth, RGB, and optical flow. A deep learning model is used for feature extraction from each stream; afterward, these features are combined with different fusion methods for the final classification. This system outperforms the results with multi-modal streams of different viewpoints collected from twelve gestures.
Noorkholis et al. [22]	In dynamic hand gesture recognition, the dataset of RGB and depth images is preprocessed from the Region of Interest (ROI) to extract the original pixel value of the hand instead of other unnecessary points. To extract the feature set, a three-dimensional convolutional neural network (3DNN) and long short-term memory (LSTM) combination of deep learning is used to extract the spatio-temporal features that are further classified by finite state machine (FSM) model classification to solve the problem of different gestures used in different applications for ease. This proposed system is designed for a smart TV environment, and for this purpose, eight gestures perform robustly in real-time testing out of 24 gestures.
K. Irie et al. [23]	In this paper, the hand gesture is detected by the emotion of the hand in front of the camera. The hand motion is detected to control the electronic appliances in intelligent rooms with complete control of hand gestures. The cameras have the ability to zoom in and focus on the user to detect the hand gesture. The hand is detected via color information and motion direction using fingers.
Chen-Chiung Hsieh et al. [24]	This research was conducted to reduce issues like hand gesture detection from complex backgrounds and light intensity issues. The hand gesture was detected with the help of the body skin detection method. The gestures were classified with the help of a new hand gesture recognition model called the motion history image-based method. A total of six hand gestures at different distances from the camera were used as the dataset. The images were trained using a haar-like structure with up, down, right, and left movements. The home automation-based system generated 94.1% accuracy using the proposed method.
Zhou Ren et al. [25]	A new study was conducted on hand gesture recognition using the finger earth mover distance (FEMD) approach. They noticed the speed and accuracy of the FEMD, shape context, and shape-matching algorithm. The dataset was collected from the Kinect camera, so it contained both depth and RGB images.
Jaya Prakash Saho [26]	Currently, convolutional neural networks (CNNs) exhibit good recognition rates for image classification problems. It is difficult to train deep CNN networks such as AlexNet, VGG-16, and ResNet from scratch due to the lack of big, labelled picture examples in static hand gesture images. To recognize hand gestures in a dataset with a low number of gesture images, they used an end-to-end fine-tuning strategy for a pre-trained CNN model with score-level fusion. They used two benchmark datasets, and the efficacy of the proposed approach was assessed using leave-one-subject-out cross-validation (LOO CV) and conventional CV tests. They proposed a real-time American Sign Language (ASL) recognition system and also evaluated it.
Ing-Jr Ding [27]	In the proposed system, the suggested method consists of two sequential computation steps: phase 1 and phase 2. The deep learning model, a visual geometry group (VGG)-type convolutional neural network (CNN), also known as the VGG-CNN, is used to assess the recognition rate. The experiments proved that image extraction efficiently eliminates the undesirable shadow region in hand gesture depth pictures and greatly improves the identification accuracy.
Jun Li [28]	They proposed MFFCNN-LSTM for forearm sEMG signal recognition using time-domain and time-frequency spectrum features. They first extracted hand movements from the NinaPro db8 dataset, and then images were denoised via empirical Fourier decomposition. The images were passed through the different channels using CNN to collect the time-domain and time-frequency-spectrum features. The features were fused and passed to the LSTM. They achieved 98.5% accuracy with the proposed system.

**Table 2:** Related work for hand gesture recognition using marker sensors.

Authors	Methodology
Safa et al. [29]	Currently, the hand gesture system deploys many recognition systems with sensors to locate the correct motion and gesture of the hand without any distortion. Combining machine learning and sensors increases the potential in the field of digital entertainment by using touchless and touch-dynamic hand motion. In a recent study, a leap motion device was used to detect the dynamic motion of the hand without touching it, analyse the sequential time series data using long short-term memory (LSTM) for recognition, and separate unidirectional and bidirectional LSTM. The novel model, named Hybrid Bidirectional Unidirectional LSTM (HBU-LSTM), improves performance by considering spatial and temporal features between leap motion data and neural network layers.
Xiaoliang et al. [30]	The hand gesture system, with a novel approach, combines a wearable armband and customized pressure sensor smart gloves for sequential hand motion. The data collected from the inertial measurement unit (IMU), fingers, palm pressure, and electromyography was computed using deep learning. Long and short-term memory models (LSTM) for testing and training were applied. The experimental work showed outstanding results with dynamic and air gestures collected from ten different participants.
Muhammad et al. [31]	In a smart home, the automatic system developed for the elder’s care deployed a home automation system with the gesture to control the appliances of daily use by using embedded hand gloves to detect the motion of the hand. For hand movements, wearable sensors such as an accelerometer and gyroscope were used to collect the combined feature set, and a random forest classifier was used to recognize the nine different gestures.
Dong-Luong-Dinh et al. [32]	In hand gesture recognition for home appliances, a novel approach towards detection is provided in this paper. They controlled home appliances using hand gestures by detecting hands and generating control commands. They created a database for hand gestures via labelling part maps and then classifying them using random forests. They generated a system for TV, lights, doors, changing channels, fans, temperature, and volume using hand gestures.
Muhammad Muneeb et al. [33]	Smart homes for the elderly and disabled people need special attention, as awareness of geriatric problems is necessary to resolve these issues. Researchers have developed many gesture recognition systems in various domains, but the authors of this paper presented a way to deal with elderly issues in particular. They used gloves to record the movements of the rotation, tilting of the hand, and acceleration. The nine gestures were classified using random forest, attaining an accuracy of 94% over the benchmark dataset.
Chi-Huang Hung et al. [34]	They proposed a system for an array lamp that performed ON/OFF actions and dimmed the light. They used a gyroscope and an accelerometer for hand detection. The noise was removed using a Kalman filter, and signals were decoded after receiving them from the devices to convert them into the desired gestures.
Marvin S. Verdadero et al. [35]	Remote control devices are common, but the setup is very expensive. The static hand gestures are taken from an Android mobile, and the signals are passed to the electronic devices. The distance should be 6 m from the device to pass the signals accurately for gesture recognition.
Zhiwen Deng [36]	Sign language recognition (SLR) is an efficient way to bridge communication gaps. SLR can additionally be used for human–computer interaction (HCI), virtual reality (VR), and augmented reality (AR). To enhance the research study, they proposed a skeleton-based self-distillation multi-feature learning method (SML). They constructed a multi-feature aggregation module (MFA) for the fusion of the features. For feature extraction and recognition, a self-distillation-guided adaptive residual graph convolutional network (SGA-ResGCN) was used. They tested the system on two benchmark datasets, WLASL and AUTSL, attaining accuracies of 55.85% and 96.85%, respectively.
Elahe Rahimian [37]	For the reduction in computation costs in complex architectures while training larger datasets, they proposed a temporal convolution-based hand gesture recognition system (TC-HGR). The 17 gestures were trained using attention mechanisms and temporal convolutions. They attained 81.65% and 80.72% classification accuracy for window sizes of 300 ms and 200 ms, respectively.

**Table 3:** Confusion matrix for gesture classification by the proposed approach using the HaGRI dataset.

Gesture Classes	Call	Dislike	Like	Mute	Ok	Stop	Two Up
call	0.93	0	0	0.03	0	0	0.04
dislike	0	0.92	0	0	0.05	0	0.03
like	0.05	0	0.95	0	0	0	0
mute	0	0.04	0	0.94	0	0.02	0
ok	0	0	0.07	0	0.93	0	0
stop	0	0.05	0	0.05	0	0.90	0
two up	0	0	0.04	0	0	0.05	0.91
Mean Accuracy = 92.57%							

**Table 4:** Confusion matrix for gesture classification by the proposed approach using the Egogesture dataset.

Gesture Classes	Scroll Hand towards Right	Scroll Hand Downward	Scroll Hand Backward	Zoom in with Fists	Zoom Out with Fists	Rotate Finger Clockwise	Zoom in with Fingers
scroll hand towards the right	0.90	0	0	0.03	0	0.07	0
scroll hand downward	0	0.93	0.07	0	0	0	0
scroll hand backward	0	0	0.92	0	0.05	0	0.03
zoom in with fists	0	0.03	0	0.93	0	0.04	0
zoom out with fists	0.04	0	0	0	0.94	0	0.02
rotate finger clockwise	0	0.07	0	0	0.02	0.91	0
zoom in with fingers	0.04	0	0	0.06	0	0	0.90
Mean Accuracy = 91.86%							

**Table 5:** Confusion matrix for gesture classification by the proposed approach using the Jester dataset.

Gesture Classes	Sliding Two Fingers Down	Stop Sign	Swiping Left	Swiping Right	Turning Hand Clockwise	Turning Hand Counterclockwise	Zoom in with Two Fingers
Sliding two fingers down	0.91	0	0	0	0	0.09	0
stop sign	0	0.92	0	0.05	0	0	0.03
swiping left	0.01	0	0.93	0	0.06	0	0
swiping right	0.06	0	0	0.92	0	0.02	0
turning hand clockwise	0	0.04	0	0	0.92	0	0.04
turning hand counterclockwise	0	0	0.08	0	0	0.92	0
zoom in with two fingers	0.06	0	0	0	0.05	0	0.89
Mean Accuracy = 91.57%							

**Table 6:** Confusion matrix for gesture classification by the proposed approach using the WLASL dataset.

Gesture Classes	Hungry	Wish	Scream	Forgive	Attention	Appreciate	Abuse
hungry	0.91	0	0	0.08	0	0	0.01
wish	0	0.90	0.09	0	0.01	0	0
scream	0.02	0.06	0.92	0	0	0	0
forgive	0	0	0	0.90	0.07	0.03	0
attention	0	0	0.04	0	0.89	0	0.07
appreciate	0	0.09	0	0.01	0	0.90	0
abuse	0.01	0	0	0	0	0.08	0.91
Mean Accuracy = 90.43%							

**Table 7:** Performance evaluation of the proposed approach using the HaGRI dataset.

Gesture Classes	Accuracy	Precision	Recall	F1 Score
call	0.98	0.93	0.95	0.94
dislike	0.97	0.92	0.91	0.92
like	0.97	0.95	0.90	0.92
mute	0.98	0.94	0.92	0.93
ok	0.98	0.93	0.95	0.94
stop	0.97	0.90	0.93	0.91
two up	0.97	0.91	0.93	0.92

**Table 8:** Performance evaluation of the proposed approach using the Egogesture dataset.

Gesture Classes	Accuracy	Precision	Recall	F1 Score
scroll hand towards the right	0.97	0.90	0.92	0.91
scroll hand downward	0.97	0.93	0.90	0.92
scroll hand backward	0.98	0.92	0.93	0.92
zoom in with fists	0.98	0.93	0.91	0.94
zoom out with fists	0.98	0.94	0.93	0.94
rotate finger clockwise	0.97	0.91	0.89	0.90
zoom in with fingers	0.98	0.90	0.95	0.92

**Table 9:** Performance evaluation of the proposed approach using the Jester dataset.

Gesture Classes	Accuracy	Precision	Recall	F1 Score
Sliding two fingers down	0.96	0.91	0.88	0.89
stop sign	0.98	0.92	0.96	0.94
swiping left	0.97	0.93	0.92	0.93
swiping right	0.98	0.92	0.95	0.93
turning hand clockwise	0.97	0.92	0.89	0.91
turning hand counterclockwise	0.97	0.92	0.89	0.91
zoom in with two fingers	0.97	0.89	0.93	0.91

**Table 10:** Performance evaluation of the proposed approach using the WLASL dataset.

Gesture Classes	Accuracy	Precision	Recall	F1 Score
hungry	0.98	0.91	0.97	0.94
wish	0.96	0.90	0.86	0.88
scream	0.97	0.92	0.88	0.90
forgive	0.97	0.90	0.91	0.90
attention	0.97	0.89	0.92	0.90
appreciate	0.97	0.90	0.89	0.90
abuse	0.97	0.91	0.92	0.91

**Table 11:** Comparison of the proposed method using conventional systems.

Methods	HaGRID	Egogesture	Jester
P. Molchanov et al. [74]	-	0.78	-
D. Tran et al. [75]	-	0.86	-
R. Cutura et al. [76]	0.89	-	-
P. Padhi [77]	0.90	-	-
J. Yang et al. [78]	-	-	0.67
S. Li et al. [79]	-	-	0.73
Proposed Method	0.92	0.91	0.91
